# 17-β estradiol exerts anti-inflammatory effects through activation of Nrf2 in mouse embryonic fibroblasts

**DOI:** 10.1371/journal.pone.0221650

**Published:** 2019-08-23

**Authors:** Chin-Hee Song, Nayoung Kim, Do-Hee Kim, Ha-Na Lee, Young-Joon Surh

**Affiliations:** 1 Department of Internal Medicine, Seoul National University Bundang Hospital, Seongnam, Gyeonggi-do, South Korea; 2 Department of Internal Medicine and Liver Research institute, Seoul National University College of Medicine, Seoul, South Korea; 3 Tumor Microenvironment Global Core Research Center, College of Pharmacy, Seoul National University, Seoul, South Korea; University of Nevada School of Medicine, UNITED STATES

## Abstract

Several reports indicate crosstalk between the transcription factor nuclear factor erythroid 2-related factor 2 (Nrf2) and estrogen, which has a protective effect in colorectal cancer (CRC). The aim of this study was to investigate the role of Nrf2 signaling in the anti-inflammatory effect of estrogen using Nrf2 knockout (Nrf2 KO) mouse embryonic fibroblasts (MEFs), a powerful system to test the function of target genes due to their easy accessibility, and rapid growth rates. After inducing inflammation by tumor necrosis factor alpha (TNF-α), the effects of 17β-estradiol (E2) on the expression of proinflammatory mediators [i.e., NF-κB and inducible nitric oxide synthase (iNOS)] and estrogen receptors were evaluated by Western blot. In wild type (WT) MEFs, E2 treatment ameliorated TNF-α-induced nuclear translocation of NF-κB and expression of its target protein iNOS. Estrogen receptor beta (ERβ) expression was decreased by TNF-α-induced inflammation and restored by E2 treatment. When treated to WT MEFs, E2 induced nuclear translocation of Nrf2. The inhibitory effect of E2 on TNF-α-induced enhancement of iNOS was markedly dampened in Nrf2 KO MEFs. Notably, ERβ expression was significantly diminished in Nrf2 KO MEFs compared to that in WT cells. Promoter Database (EPD) revealed two putative anti-oxidant response elements (AREs) within the mouse ERβ promoter. Furthermore, in WT MEFs, E2 treatment repressed TNF-α-induced expression of iNOS protein and recovered by 4-(2-phenyl-5,7-bis(trifluoromethyl)pyrazolo(1,5-a)pyrimidin-3-yl)phenol (PHTPP), a selective ERβ antagonist, treatment, but not in Nrf2 KO MEFs. In conclusion, Nrf2 plays a pivotal role in the anti-inflammatory of estrogen by direct regulating the expression of ERβ.

## Introduction

Inflammation in which immune cells, blood vessels, and molecular mediators are involved [1] is generally classified as either acute or chronic. Chronic inflammation plays a key role in a variety of inflammatory diseases, including carcinogenesis [2], rheumatoid arthritis [3], and neurodegenerative diseases [4]. To prevent the inflammation, our body tries to localize and eliminate the initial cause of tissue injury and to remove damaged tissue components, which facilitate tissue repair.

The complex immunomodulating role of estrogen on the inflammatory process [5,6] revealed that estrogens affect susceptibility to chronic inflammatory diseases and response to infections in relation to the menstrual cycle, pregnancy, and menopause [5]. Interestingly, a large number of data suggest a protective role of estrogen in chronic inflammatory diseases, such as neurodegenerative diseases [7] and colon cancer development [8–10]. Furthermore, estrogen suppressed inflammation in rat cerebral blood vessels by blocking the interleukin (IL)-1β-mediated induction of the NF-κB/cyclooxygenase-2 (COX-2) pathway [11]. These diverse and contradictory results are attributable, at least in part, presence of two different types of receptors, estrogen receptor alpha (ERα) and beta (ERβ) encoded by distinct genes which have differential expression patterns between tissues and organs [12]. In particular, ERβ is closely related to the protective functions of estrogen. In human colon tissue, ERβ was predominantly expressed in normal colonic mucosa, but its expression level was decreased in colon cancers. In contrast, ERα expression was found to be enhanced in colon cancer compared to normal colon tissue [13]. In female normal colonic epithelial CCD841CoN cells, selective ERβ antagonist, 4-(2-phenyl-5,7-bis(trifluoromethyl)pyrazolo(1,5-a)pyrimidin-3-yl)phenol (PHTPP), abrogated the inhibitory effect of 17β-estradiol (E2) on the tumor necrosis factor alpha (TNF-α)-induced COX-2 expression [14]. Furthermore, compared to wild type mice, ERβ knockout (KO) mice showed more severe clinical symptoms, such as colon shortening, elevated inflammation score, grade of dysplasia, and the greater number and the size of polyps [15]. In addition, the mRNA expression of inflammatory genes, including IL-6, IL-17, TNF-α, and interferon-gamma (IFNγ), was significantly increased in ERβ KO mice [15].

Nuclear factor erythroid 2-related factor 2 (Nrf2) is a well-known transcription factor that regulates an adaptive cellular defense response to various stresses, including oxidative, proteotoxic, and metabolic stresses as well as inflammation [16]. Under stress conditions, Nrf2 regulates downstream target genes encoding antioxidant and phase II carcinogen detoxifying enzymes, such as heme oxygenase-1 (HO-1), nicotinamide adenine dinucleotide phosphate: quinone dehydrogenase 1 (NQO1), glutamate-cysteine ligase catalytic subunit (GCLC), and glutamate-cysteine ligase modifier subunit (GCLM), through binding to the consensus binding sequence (5’-TGACnnnGC-3’) called the antioxidant response element (ARE) [17]. The activation of these enzymes potentiates antioxidant capacity of the cells, thereby protecting against diseases that are often associated with oxidative stress [18,19]. It has been reported that the activation of Nrf2 prevents the expression of proinflammatory cytokines, including IL-1β, IL-6, and IL-17, in lipopolysaccharide (LPS)-induced macrophages [20], dextran sodium sulfate (DSS)-induced murine colitis [21], and experimentally induced autoimmune encephalomyelitis in mice [22]. Nrf2 has also been reported to inhibit inflammation by attenuating the NF-κB signaling through up-regulation of HO-1 [23,24].

Gene KO models are commonly used to study the function of genes. Instead of *in vivo* KO animal experiments, *ex vivo* culture of mouse embryonic fibroblasts (MEFs) prepared from mouse embryos represents a powerful system to test the function of target genes due to their easy accessibility, rapid growth rates, and possibility of a large number of experiments [25]. MEFs are often used as “feeder cells” that help to support and maintain mouse and human embryonic stem cells in an undifferentiated state [26]. Additionally, MEFs can be converted to a pluripotent state or directly transdifferentiated into mature cells, such as functional neurons [27] and cardiomyocytes [28]. Because of their ubiquitous distribution as tissue cells, the MEFs are poised to respond to factors released by newly activated innate immune cells, and hence often used in studying inflammation and immunity [25].

Previously, we reported that E2 (10 mg/kg) inhibited the initiation of colorectal cancer (CRC) by activating Nrf2 in azoxymethane (AOM) plus DSS-treated male ICR mice, which showed more severe colitis-associated colon carcinogenesis compared to female mice [9]. Furthermore, the protective effect of E2 on the intestinal barrier was mediated by inducing the expression of MUC2 and tight junction molecules (i.e., ZO-1, OCLN, and CLDN4) and inhibiting proinflammatory cytokines (i.e., NF-κB and COX-2) in male ICR mice treated with AOM and DSS [29]. We further verified that E2 exerted anti-inflammatory effects by blocking NF-κB signaling and downregulating COX-2 expression and inducing expression of anti-oxidant enzymes and ERβ in CCD841CoN cells [14]. Based on this background information, we hypothesized that E2 inhibits inflammation through directly activating Nrf2. To test this hypothesis, we explored the expression profiles of proinflammatory mediators modulated by E2 in MEFs prepared from Nrf2 knockout (Nrf2^−/−^) and wild type (Nrf2^+/+^) mice.

## Materials and methods

### Preparation and culturing of mouse embryonic fibroblasts and treatment

MEFs were isolated from Nrf2 knockout (Nrf2^−/−^) and wild type (Nrf2^+/+^) mice on a C57BL6/129SV mixed background generated by the laboratory of Yuet Wai Kan [30] which were kindly by Professor Jeffrey Johnson (University of Wisconsin-Madison). Nrf2 knockout and WT MEFs were a kind gift from Prof. Young-Joon Surh, Seoul National University. Briefly, the Nrf2^−/−^, Nrf2^+/−^, and wild-type mice were maintained in the animal quarters in accordance with the university guidelines for Seoul National University Animal Care and Use committee (SNU 20140624–2) and were housed in a 12 h light/dark cycle. They were fed standard rodent chow and given water ad libitum. Male and female Nrf2^+/−^ mice were paired and the pregnancies were monitored. Embryos were obtained at the day 13.5 after pairing under aseptic conditions. The heads of the embryos were used to confirm the Nrf2 genotype by PCR, and the embryo bodies were minced into small pieces and cultured in high glucose Dulbecco’s Modified Eagle’s Media (DMEM) supplemented with 10% fetal bovine serum (FBS) and kept at 37°C with 5% CO_2_ [31].

The cells were maintained in DMEM supplemented with 10% FBS and antibiotic-antimycotic mixture (Gibco BRL, Gaithersburg, MD, USA). The cells were kept in phenol red-free DMEM supplemented with 10% charcoal-stripped FBS (CSS) for 24 h for serum starvation prior to stimulation. This step is necessary to maintain the quiescent status of these cells before they are exposed to proinflammatory stimuli for optimal responses [25]. The cells were then treated with or without 10 ng/mL human recombinant TNF-α for 6 h (210-TA-005, R&D system, Minneapolis, MI, USA) in the absence or presence of 10 nM water soluble E2 (Sigma E4389, Sigma-Aldrich Co., St. Louis, MO, USA) for 48 h. In addition, 10 μM PHTPP (Sigma SML1355, Sigma-Aldrich Co., St. Louis, MO, USA) was treated for 48 h in the presence of E2. All cells were cultured at 37°C in a 95% humidified atmosphere containing 5% CO_2_.

### Western blot analysis

After treatment, the media on the apical side were aspirated, and the cells were collected by centrifugation at 1,000 rpm for 5 minutes at 4°C. Cells were suspended in RIPA cell lysis buffer (Cell Signaling Technology, Beverly, MA, USA) with protease and phosphatase inhibitors and kept on ice for 20 minutes. After centrifugation at 13,000 rpm for 15 minutes, the supernatant was collected and stored at -70°C until use.

Whole cell extracts were isolated using RIPA buffer with protease and phosphatase inhibitors. Protein concentrations were determined using the bicinchoninic acid (BCA) protein assay reagent (Pierce). Protein samples were mixed with an equal volume of 5 × sodium dodecyl sulfate (SDS) sample buffer, boiled for 5 minutes, and then separated using 8% to 15% SDS-polyacrylamide gel electrophoresis (PAGE). After electrophoresis, proteins were transferred to polyvinylidene difluoride (PVDF) membranes. The membranes were blocked with 5% nonfat dry milk in Tris-buffered saline with Tween-20 buffer (TBST) for 1 h at room temperature. Membranes were incubated overnight at 4°C with specific antibodies. Primary antibodies were removed by washing the membranes three times in TBS-T and incubated for 2 h with horseradish peroxidase-conjugated anti-rabbit, anti-goat, or anti-mouse immunoglobulin antibody (Santa Cruz Biotechnology, Dallas, TX, USA). Following three washes with TBS-T, antigen-antibody complexes were detected with the ECL Prime Western Blotting Detection Kit (GE Healthcare Biosciences, Piscataway, NJ, USA). The intensity of the blots was quantified by densitometry analysis using ImageJ software (National Institutes of Health, Bethesda, MD, USA). The antibodies are listed in detail in Table 1.

**Table 1:** List of antibodies and their characteristics.

Antigen	Antibody	Dilution
Nrf2	Abcam (ab62352)	WB (1:500)
HO-1	Abcam (ab13248)	WB (1:500)
NF-κB p65	Santa Cruz Biotechnology (sc8008)	WB (1:500)
iNOS	BD Biosciences (#610328)	WB (1:1000)
ERα	Abcam (ab93021)	WB (1:500)
ERβ	Abcam (ab3576)	WB (1:500)
Lamin B	Santa Cruz Biotechnology (sc6216)	WB (1:1000)
β-actin	Santa Cruz Biotechnology (sc47778)	WB (1:3000)

WB, Western blot; Nrf2, nuclear factor erythroid 2-related factor 2; HO-1, heme oxygenase 1; NF-κB, nuclear factor-kappa B; iNOS, inducible nitric oxide synthase; ER, estrogen receptor; β-Actin, beta-actin.

### Preparation of nuclear protein fractions

The NE-PER Nuclear and Cytoplasmic Extraction Kit (Pierce, Rockford, IL, USA) was used for efficient cell lysis and extraction of cytoplasmic and nuclear protein fractions according to the manufacturer’s instructions. The localization of Nrf2 and NF-κB into the nucleus was analyzed using Western blotting. To ensure proper separation of the subcellular fractions, an anti-Lamin B-specific antibody against nuclear fractions was used.

### Identification of Nrf2-binding sites through *in silico* analysis

The JASPAR CORE database [32] and Eukaryotic Promoter Database (EPD) [33] were used to identify the Nrf2 binding sites, called ARE, within the promoter region of the ERβ gene. JASPAR is a collection of transcription factor DNA-binding preferences used for scanning genomic sequences [32]. EPD is a biological database and web-based tool to predict putative transcription factor binding elements of eukaryotic RNA polymerase II promoters with experimentally defined transcription start sites (TSSs) [33]. In EPD, the search was limited to the -5 kb upstream promoter region relative to TSS. In this study, we focused on the proximal promoter region within the -1 kb upstream promoter of TSS.

### Statistical analysis

Data are expressed as the mean ± SEM. Statistical significance was examined with the Mann-Whitney U test. A p-value < 0.05 was considered to indicate statistical significance. All statistical analyses were conducted using GraphPad Prism (GraphPad, La Jolla, CA, USA) and SPSS version 18.0 (SPSS Inc., Chicago, IL, USA).

## Results

### The expression and activation of Nrf2 were completely abolished in Nrf2 KO MEFs

**Fig 1:**
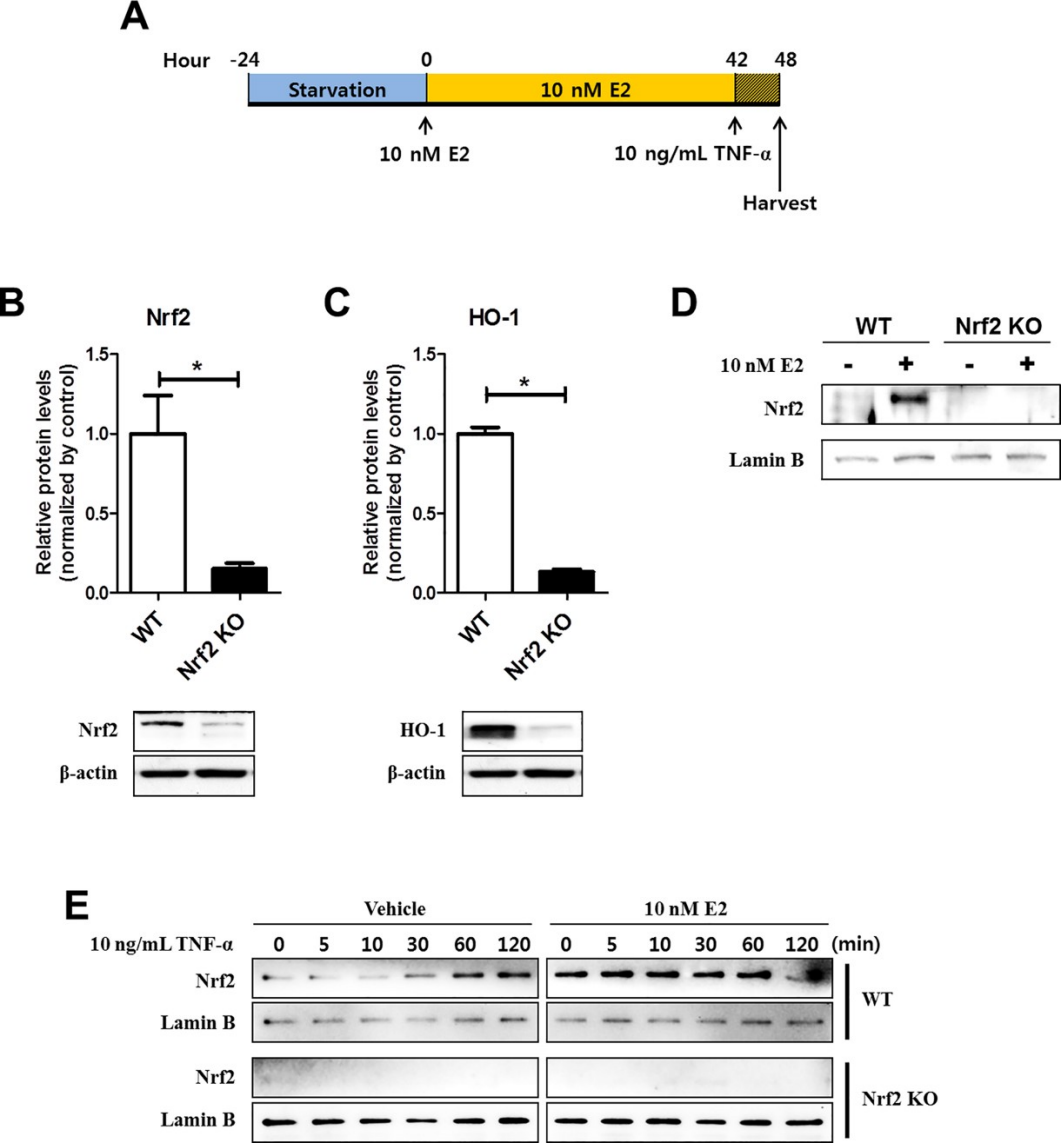
The protein expression and activation of Nrf2 in WT and Nrf2 KO MEFs. (A) The experimental scheme to evaluate the effects of E2 against TNF-α-induced inflammation in WT and Nrf2 KO MEFs. (B-C) WT and Nrf2 KO MEFs were kept in phenol red-free DMEM supplemented with 10% CSS for 3 days and harvested for Western blot analysis to measure expression of Nrf2 (B) and HO-1 (C). (D) WT and Nrf2 KO MEFs were treated with or without 10 nM E2 for 48 h and harvested for Western blot analysis to measure protein expression of nuclear Nrf2. (E) WT and Nrf2 KO MEFs were treated with 10 ng/mL TNF-α for the indicated times in the absence or presence of 10 nM E2 for 48 h followed by Western blot analysis with Nrf2 antibody. β-actin and Lamin B were used as internal controls to normalize the expression. Mean with SEM. *, p<0.05 for comparison between two groups.

MEFs from Nrf2 WT or KO mice were subjected to serum starvation for 24 h to keep the quiescent status for optimal response [14,25]. Cells were then treated with 10 nM of E2 or vehicle for 42 h, followed by treatment of TNF-α (10 ng/mL) for 6 h (Fig 1A) as described in our previous study [14].

To verify the Nrf2 deficiency in Nrf2 KO MEFs, the expression of Nrf2 was measured. As shown in Fig 1B, the total protein expression of Nrf2 was strongly abolished in Nrf2 KO MEFs compared to WT cells (p = 0.03 for WT vs Nrf2 KO). In line with this observation, the expression of HO-1, one of the representative target protein of Nrf2, was almost completed suppressed in Nrf2 deficient MEFs (Fig 1C, p = 0.03 for WT vs Nrf2 KO). Next, the protein expression levels of nuclear Nrf2 in Nrf2 WT and KO MEFs were measured by Western blot analysis. In WT MEFs, the nuclear translocation of Nrf2 increased by E2 treatment which was not achievable in Nrf2 KO MEFs (Fig 1D). As shown in Fig 1E, under TNF-α only treatment, the level of translocated Nrf2 to the nucleus at early time points (5 and 10 min) showed similar to basal level (0 min). After 30 min treatment with TNF-α, Nrf2 was translocated to the nucleus in a time-dependent manner. However, under E2 treated condition, the nuclear translocated Nrf2 consistently kept at high level by 60 min even under TNF-α treatment (Fig 1E).

### E2 failed to inhibit the proinflammatory protein expression in TNF-α-treated Nrf2 KO MEFs

**Fig 2:**
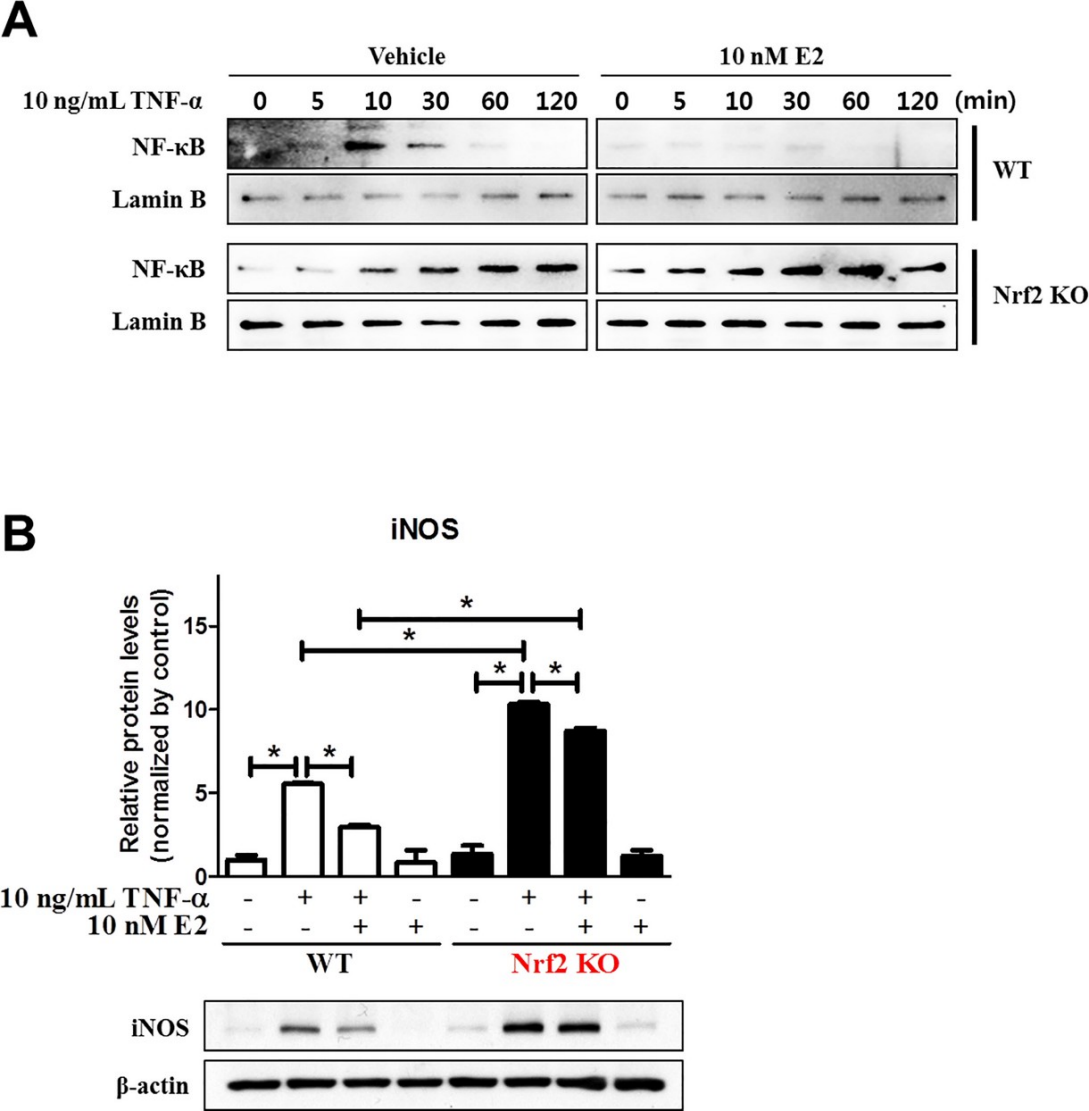
Inhibitory effect of E2 on the TNF-α-induced expression of nuclear NF-κB and iNOS through Nrf2. (A) WT and Nrf2 KO MEFs were treated with 10 ng/mL TNF-α for the indicated times in the absence or presence of 10 nM E2 for 48 h followed by Western blot analysis with NF-κB antibody. (B) WT and Nrf2 KO MEFs were treated with 10 ng/mL TNF-α for 6 h in in the absence or presence of 10 nM E2 for 48 h and harvested for Western blot analysis to measure expression of iNOS. Lamin B and β-actin were used as internal controls to normalize the expression. Mean with SEM. *, p<0.05 for comparison between two groups.

To investigate whether estrogen modulates expression of inflammatory factors through activation of Nrf2, we measured expression levels of the prototypic proinflammatory transcription factor NF-κB and its target protein iNOS using Western blot analysis. In WT MEFs, TNF-α treatment transiently increased the expression of nuclear NF-κB, which peaked at 10 min, while pre-treatment with E2 completely blocked TNF-α-induced upregulation of nuclear NF-κB (Fig 2A). Interestingly, the protein expression level of nuclear NF-κB gradually increased by TNF-α treatment in a time-dependent manner in Nrf2 KO MEFs in both E2 pretreated and untreated conditions (Fig 2A). Next, we measured the protein expression level of iNOS which is a well-known NF-κB target protein. In WT MEFs, TNF-α treatment induced iNOS expression (p = 0.05 for vehicle vs TNF-α), and this was attenuated by E2 pretreatment (p = 0.05 for TNF-α vs TNF-α plus E2) (Fig 2B). The lack of Nrf2 resulted in a significant increase in TNF-α-induced iNOS expression (p = 0.05 for vehicle vs TNF-α), and the inhibitory effect of E2 on TNF-α-induced iNOS expression was also diminished in the absence of Nrf2 (p = 0.05 for TNF-α vs TNF-α plus E2) (Fig 2B).

### ERβ expression, but not ERα, was completely abolished in Nrf2 KO MEFs

**Fig 3:**
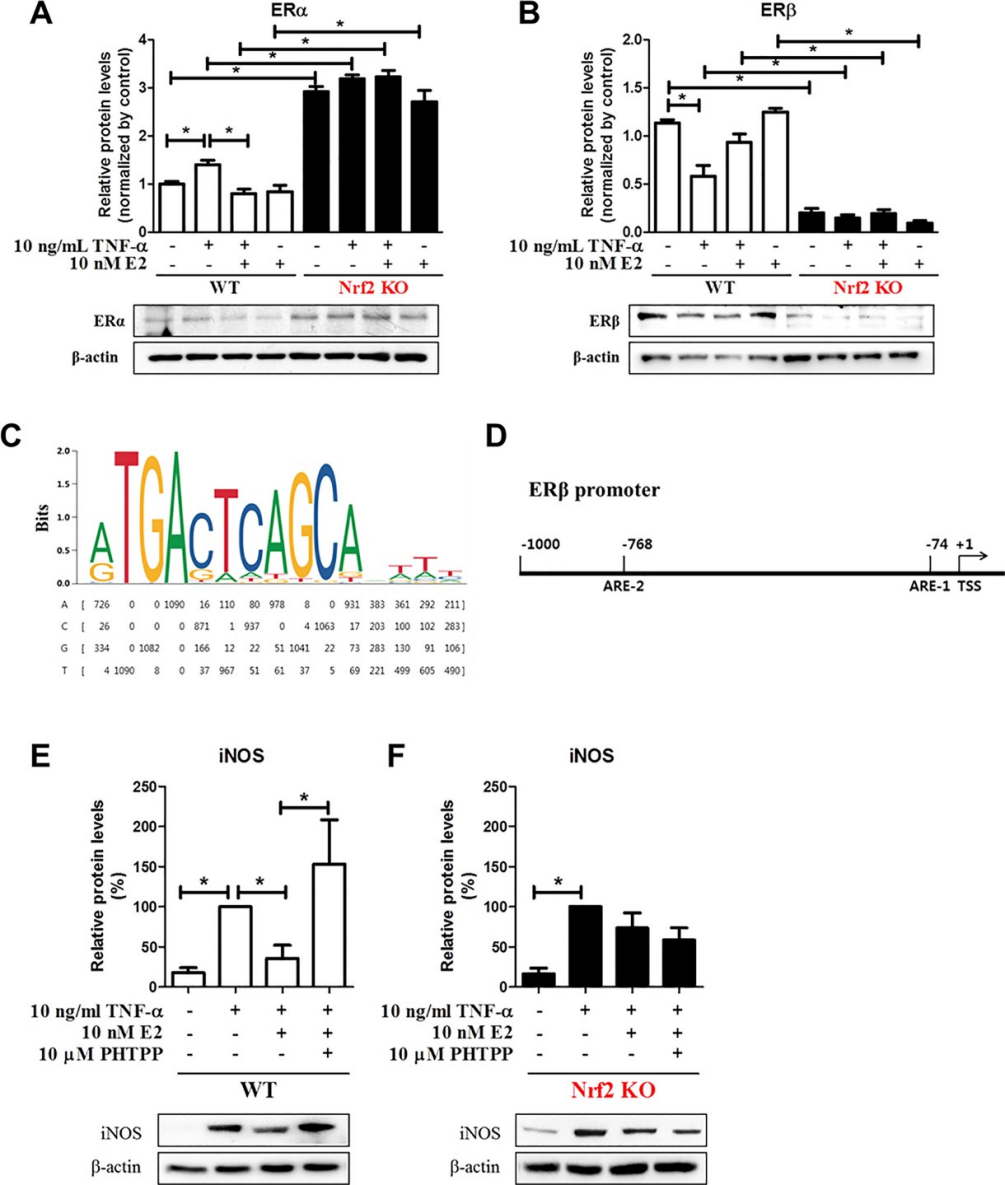
The protein expression of ERα and ERβ was oppositely altered in WT and Nrf2 KO MEFs. (A-B) WT and Nrf2 KO MEFs were treated with 10 ng/mL TNF-α for 6 h in in the absence or presence of 10 nM E2 for 48 h and harvested for Western blot analysis to measure expression of ERα (A) and ERβ (B). (C) A MAF/NF-E2 complex binding motif logo (upper panel) and frequency matrix (bottom panel) from JASPAR. (D) *In silico* analysis of Nrf2 binding sites. Schematic representation shows two putative positions of *in silico-*predicted Nrf2 binding sites (AREs) in the promoter region of mouse ERβ. (E-F) WT (E) and Nrf2 KO MEFs (F) were treated with 10 ng/mL TNF-α for 6 h in the absence or presence of 10 nM E2 for 48 h. 10 μM PHTPP was treated along with E2 for 48 h and harvested for Western blot analysis to measure expression of iNOS. β-actin was used as an internal control to normalize the expression. Mean with SEM. *, p<0.05 for comparison between two groups.

To further evaluate the effects of E2 on the mutual regulation between Nrf2 and ERs, we measured the protein expression levels of ERα and ERβ in WT and Nrf2 KO MEFs. In WT MEFs, the expression level of ERα was decreased by E2 treatment (p = 0.01 for TNF-α vs TNF-α plus E2) (Fig 3A). However, ERα expression strongly increased in Nrf2 KO MEFs even at the basal expression level (Fig 3A). In contrast, the protein expression level of ERβ, which is highly expressed at the basal level, was inhibited by TNF-α treatment in WT MEFs (p = 0.01 for vehicle vs TNF-α). The decrease was recovered by E2 treatment (p = 0.07 for TNF-α vs TNF-α plus E2) (Fig 3B). Surprisingly, ERβ expression was almost completely abolished in Nrf2 KO MEFs (Fig 3B). To explain the regulatory mechanism, we performed *in silico* promoter analysis for the Nrf2 binding site sequence on the ERβ promoter using the JASPAR CORE database and EPD web-based tools (Fig 3C and 3D). We found the MAF/NF-E2 binding motif (5’-rTGACTCAGCArwwy-3’), which contains the core consensus ARE sequence (5’-TGACnnnGC-3’) [34], from the JASPAR 2018 library (Name: MAF::NFE2, matrix ID: MA0501.1, and data type: ChIP-seq) [32]. Through *in silico* promoter analysis using the EPD website (https://epd.vital-it.ch/index.php), we were able to identify two putative AREs within the -1 kb upstream promoter of the TSS of the mouse ERβ gene (ENSEMBL ID: ENSMUSG00000021055 or RefSeq ID: NR_104386) (Fig 3D). To further verify the function of ERβ on TNF-α-mediated inflammation, WT and Nrf2 KO MEFs were treated with ERβ-specific antagonist PHTPP. In WT MEFs, TNF-α-mediated enhanced expression level of iNOS protein was approximately 64% decreased by E2 treatment (p = 0.04 for TNF-α vs TNF-α plus E2) and the expression was strongly recovered by PHTPP treatment (p = 0.05 for TNF-α plus E2 vs TNF-α plus E2 and PHTPP) (Fig 3E). However, in Nrf2 KO MEFs, TNF-α-mediated increased expression level of iNOS protein was approximately 26% decreased by E2 treatment, but not significantly, and the expression was not changed by PHTPP treatment (Fig 3F).

## Discussion

Several reports indicate crosstalk between Nrf2 and estrogen. However, most of these studies examined the effect of estrogen on the Nrf2 signaling [35–37]. Our present study demonstrates, for the first time, that Nrf2 plays a pivotal role in the anti-inflammatory effects of estrogen, and this is attributable to its upregulating the expression of ERβ through promoter binding. In order to precisely assess the involvement of Nrf2 in mediating the estrogen effects, we used MEFs prepared from WT and Nrf2 KO mice. Our results indicate that E2 acts through the Nrf2 pathway to reduce inflammation, as demonstrated by its downregulation of proinflammatory protein expression to a much greater extent in WT MEFs than in Nrf2 KO MEFs. Inflammation-mediated reduction of ERβ expression was also recovered by E2 treatment. One of the most salient features of our present study is that ERβ expression was diminished in Nrf2 KO MEF cells. Furthermore, we identified two putative core ARE motifs within the mouse ERβ promoter through *in silico* analysis. Also, the anti-inflammatory function of ERβ was confirmed by a selective ERβ antagonist PHTPP treatment. These results suggest that Nrf2 plays a critical role in mediating the anti-inflammatory effects of E2, which is likely to be mediated through the ERβ signaling.

TNF-α is one of the most potent proinflammatory cytokines that that triggers the inflammatory response [38]. NF-κB activation is essential for the TNF-α-induced inflammatory response [39]. The development of chronic inflammatory diseases, including rheumatoid arthritis, multiple sclerosis, inflammatory bowel disease, and ulcerative colitis, is associated with NF-κB overactivation [40]. In the present study, E2 treatment inhibited iNOS expression as well as NF-κB activation in TNF-α stimulated WT MEFs. Notably, inhibition of NF-κB activation by E2 appeared only in the early treatment with E2. However, the anti-inflammatory effect of E2 was weakened in Nrf2 KO MEFs. Numerous studies have demonstrated the anti-inflammatory effects of estrogen [7,41]. In addition, Boyanapall et al. reported that peritoneal macrophages derived from Nrf2 KO mice are less responsive to the anti-inflammatory effects of phenethyl isothiocyanate and curcumin in terms of inhibition of proinflammatory protein (i.e., COX-2, iNOS, IL-6, and TNF-α) and induction of anti-oxidant enzyme (i.e., HO-1) expression [42]. As Nrf2 counteracts the NF-κB [43], the anti-inflammatory effect of E2 appears to be associated with its potentiation of Nrf2 signaling though its direct inhibition of up-/down-stream of NF-κB signaling cannot be excluded.

The Nrf2 signaling pathway plays a key role in the regulation of inflammation and oxidative stress both *in vitro* [42,44,45] and *in vivo* [46,47]. It has been well-documented that Nrf2 activation leads to increased expression of the antioxidant enzyme HO-1, which also exerts anti-inflammatory effects by inducing IL-10, an anti-inflammatory cytokine, in mouse liver, human HepG2 cells, and mouse J774.1 macrophages [48]. It has been shown that the lack of Nrf2 in sickle cell disease (SCD) mice produced greater splenomegaly with red pulp expansion and obscured architecture [49]. In addition, SCD-Nrf2 KO mice exhibited reduced expression of its target antioxidant proteins, leading to increased levels of ROS, proinflammatory cytokines, and adhesion molecules [49]. Furthermore, we proposed regulatory mechanism of estrogen during colitis and colon cancer progression in terms of Nrf2 in AOM/DSS-treated mouse model [9]. Following our previous results, estrogen activated Nod-like receptor protein 3 (NLRP3) inflammasome complexes, which are inducing pyroptosis to eliminate precancerous cells, via Gα13-protein kinase Cδ (PKCδ)-Nrf2 signaling pathway. After then, NF-κB was inhibited by Nrf2 and anti-oxidant enzymes. In contrast, in the absence of estrogen, NF-κB pathway was activated by inflammatory stimuli. However, after unsuccessful elimination of precancerous cells, inflammation progressed to cancer via Gα12/Gα13-NF-κB pathway and Gα13-PKCδ-Nrf2 signaling pathway. Nrf2 promoted tumor progression by activation of anti-oxidant enzymes and NLRP3 inflammasome complexes. Numerous studies have suggested that Nrf2-deficient mice are more susceptible to inflammatory disorders [50,51]. For instance, Nrf2 knockout enhanced intestinal tumorigenesis in adenomatous polyposis coli *APC*^min/+^ mice due to attenuation of the anti-oxidative stress defense with concomitant exacerbation of inflammation [52]. In the present study, the level of iNOS protein expression was approximately 47% and 15% significantly inhibited by 10 nM E2 treatment in TNF-α stimulated WT MEFs and Nrf2 KO MEFs, respectively. However, the expression level of iNOS significantly decreased by 10 nM E2 treatment was significantly higher in Nrf2 KO than in WT. Study on peritoneal macrophages prepared from WT and Nrf2 KO mice have also reported the anti-inflammatory effects of phytochemicals such as phenethyl isothiocyanate and curcumin via Nrf2 [42]. According to their findings, iNOS protein expression was inhibited by phenethyl isothiocyanate and curcumin treatment both in WT and Nrf2 KO macrophages. Additionally, the presence of Nrf2 has more attenuating effects on COX-2 and iNOS [42]. Furthermore, in the present study, the TNF-α-induced activation of NF-κB was gradually increased in a time dependent manner by Nrf2 deficiency compared to WT MEFs. Moreover, the inhibitory effect of E2 on the expression of iNOS and activation of NF-κB was weakened in Nrf2 KO MEFs. Taken together, these data suggest that Nrf2 plays a major role in mediating the anti-inflammatory properties of estrogen. To overcome the limitation of our *ex vivo* study, we will perform further animal experiments using Nrf2 knockout mice to clarify the regulation of the NF-κB signaling pathway through Nrf2.

**Fig 4:**
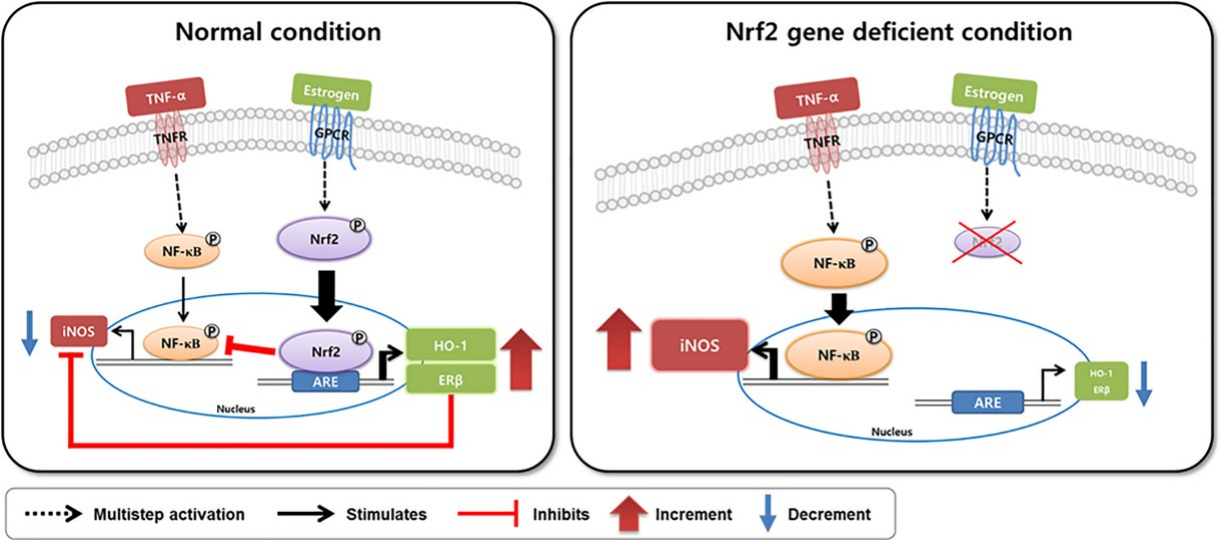
Proposed scheme illustrating the regulatory mechanism of estrogen via Nrf2 and ERβ against TNF-α mediated inflammation. In TNF-α-treated WT MEFs, estrogen inhibits expression of inflammatory signaling molecules including nuclear NF-κB and iNOS (left panel). However, in TNF-α-treated Nrf2 KO MEFs, estrogen failed to inhibit expression of nuclear NF-κB and iNOS (right panel).

In conclusion, when compared to the effect of estrogen in TNF-α-treated WT MEFs, estrogen failed to inhibit expression of inflammatory signaling molecules including nuclear NF-κB and iNOS in Nrf2 KO MEFs as schematically illustrated in Fig 4. Our data suggest that Nrf2 acts as a key player in the anti-inflammatory effects of estrogen. Furthermore, our data suggest that Nrf2 could directly regulate the expression of ERβ by binding to the ARE consensus sequence within the ERβ promoter.
